# Long-Term Results of Serial Exercise Testing and Echocardiography Examinations in Patients with Pulmonary Stenosis

**DOI:** 10.3390/jcdd10010031

**Published:** 2023-01-16

**Authors:** Chia-Hsin Yang, Li-Yun Teng, Ming-Wei Lai, Ken-Pen Weng, Sen-Wei Tsai, Ko-Long Lin

**Affiliations:** 1 Department of Physical Medicine and Rehabilitation, Taichung Tzu Chi Hospital, Taichung 427213, Taiwan; 2 School of Medicine, Tzu Chi University, Hualien 970374, Taiwan; 3 School of Medicine, College of Medicine, China Medical University, Taichung 404328, Taiwan; 4 Congenital Structural Heart Disease Center, Department of Pediatrics, Kaohsiung Veterans General Hospital, Kaohsiung 813414, Taiwan; 5 School of Medicine, College of Medicine, National Yang Ming Chiao Tung University, Taipei 112304, Taiwan; 6 School of Medicine, College of Medicine, Kaohsiung Medical University, Kaohsiung 807378, Taiwan; 7 Department of Physical Medicine and Rehabilitation, Kaohsiung Veteran General Hospital, Kaohsiung 813414, Taiwan

**Keywords:** valvular heart disease, pulmonary hypertension, cardiopulmonary exercise testing, sports cardiology, rehabilitation

## Abstract

Pulmonary stenosis (PS) affects cardiopulmonary function and exercise performance. Cardiopulmonary exercise testing (CPET) together with transthoracic echocardiography (TTE) can measure exercise performance, PS progression, and treatment effects. We assessed exercise capacity in PS patients using these methods. We enrolled 28 PS patients aged 6–35 years who received surgery, balloon pulmonary valvuloplasty, and follow-up care. The control population was selected by a 1:1 matching on age, sex, and body mass index. Baseline and follow-up peak pulmonary artery pulse wave velocity (PAV) were compared using TTE. Initial CPET revealed no significant differences in anaerobic metabolic equivalent (MET), peak oxygen consumption (VO_2_), and heart rate recovery between the two groups, nor were significant differences in pulmonary function identified. Within the PS group, there were no significant differences in MET, peak VO_2_, and heart rate recovery between the baseline and final CPET. Similarly, no significant differences were observed between the baseline and final PAV. The exercise capacity of patients with properly managed PS was comparable to that of healthy individuals. However, during the follow-up, declining trends in pulmonary function, aerobic metabolism, and peak exercise load capacity were observed among adolescents with PS. This study provides long-term data suggesting that PS patients should be encouraged to perform physical activity. Regular reevaluation should also be encouraged to limit performance deterioration.

## 1. Introduction

Pulmonary stenosis (PS) is the narrowing of the valve located between the right ventricle and pulmonary arteries. It is one of the most common congenital heart diseases (CHD); it reduces blood flow through the valve and can be seen in children [1,2]. PS affects 1 out of 2000 live births and accounts for 8% of all CHD cases. An echocardiography helps determine the structure of the pulmonary valve (PV) and quantify disease severity. A quantitative assessment of PS severity is primarily dependent on the transpulmonary pressure gradient (TPG). The TPG was found to be <36 mmHg (peak velocity < 3 m/s) in mild PS, 36–64 mmHg (peak velocity 3–4 m/s) in moderate PS, and >64 mmHg (peak velocity > 4 m/s) in severe PS [3]. The severity of PS determines the need and timing of appropriate intervention and conservative management [4,5].

Patients with mild PS show no symptoms of valve disease. Generally, adult patients with mild PS show no progression of stenosis, and most patients have a continuously high functional status. Numerous patients, especially symptomatic patients, with moderate PS receive definitive therapy in childhood. Severe PS mostly occurs in childhood and is characterized by right ventricular failure and cyanosis. Regardless of symptomatology, adults with severe PS usually receive early treatment with surgery or balloon pulmonary valvuloplasty (BPV) [3]. After successful treatment, the prognosis remains positive in adulthood [6,7,8].

Clinicians restrict patients with CHD from performing activities because the dangers of increased activity cannot be overlooked. Nevertheless, it is commonly believed that promoting physical activity is beneficial to the health and well-being of children and adults with CHD [9]. Cardiopulmonary exercise testing (CPET) is the gold standard method for evaluating exercise capacity to formulate recommendations based on the clinical status of individual patients. CPET provides noninvasive, dynamic measurements that can be used for diagnostic, prognostic, and evaluative purposes. However, in the existing studies, there is a lack of data regarding longitudinal examinations of exercise capacity in patients with PS. During progressive CPET, the measurement of maximum oxygen consumption (VO_2_ max) is universally accepted for aerobic fitness assessment [10]. Patients suffering from decreased peak VO_2_ have a higher risk of heart failure and sudden death [11].

In this study, we aimed to examine the functional results of PS patients undergoing appropriate management using serial follow-up CPET. Furthermore, we aimed to detail the effects of PS on patient exercise capacity.

## 2. Materials and Methods

### 2.1. Patient Selection and Data Collection

We retrospectively examined the medical records of patients with isolated PS, which were obtained from the Pediatric Outpatient Department of Kaohsiung Veteran General Hospital in Taiwan. Thirty patients with PS were enrolled in this study between December 2013 and January 2022. The study included patients who were aware of the steps involved in treadmill exercise testing and could complete the test without demonstrating abnormal electrocardiographic findings or symptoms. Patients with other CHDs (e.g., ventricular septal defect, atrial septal defect, and patent ductus arteriosus) and those with current or a history of arrhythmia were excluded. All patients had a typical form of PS. Patients who underwent surgery or BPV had severe or moderate PS with progressive symptoms while those who received follow-up care had mild or asymptomatic moderate PS. Current guidelines were applied while managing the patients [3]. After body composition measurements, all patients underwent CPET and pulmonary function tests.

To facilitate the comparisons of the selected patient CPET parameters, a control population was chosen from a database of healthy individuals undergoing CPET at the Veteran General Hospital of Kaohsiung; this population had no cardiac anomalies or underlying diseases. The control population was matched with the patient population in a 1:1 ratio in terms of age, sex, and body mass index (BMI) using MedCalc (version 14.12.0; MedCalc, Ostend, Belgium). Informed consent was obtained from all the patients’ parents before the examinations.

### 2.2. Treadmill Exercise Testing

The American College of Sports Medicine (ACSM) [12] has indicated that treadmill exercise testing can be conducted from three years of age. All patients underwent symptom-limited CPET involving a treadmill, a flow module, a gas analyzer, and an electrocardiographic monitor (Metamax 3 B; Cortex Biophysik GmbH Co., Liepzig, Germany); the Bruce protocol was adopted. During testing, oximetry, blood pressure, and heart rate (HR) were monitored. The ACSM [13] has indicated that the test should be terminated when the patient exhibits unbearable symptoms, can no longer proceed, or has performed the maximum amount of exercise possible. Oxygen consumption (VO_2_) was measured using a breath-by-breath method.

The metabolic equivalent (MET) value (i.e., 3.5 mL of oxygen per kilogram of body mass per minute [14]) was measured after VO_2_. The peak and anaerobic threshold (AT) of the MET were described as the maximum MET and MET at AT throughout the exercise test, respectively. The AT was based on the ventilatory equivalents of the oxygen ratio (i.e., expired volume [VE]/VO_2_) and ventilation/carbon dioxide production slope (i.e., VE/volume of exhaled carbon dioxide [VCO_2_]) [15]. The respiratory gas exchange ratio (RER) was calculated as VCO_2_/VO_2_. HR recovery (HRR) was calculated by subtracting the HR measured a minute after testing from the maximum HR measured throughout the test. VO_2_ max was defined under three circumstances: the RER was >1.1, the peak HR was >200 bpm, or the HR was >85% of the age-predicted maximum [12,16,17].

### 2.3. Pulmonary Function Testing

Pulmonary function tests were conducted through spirometry at rest and included measurements of the forced vital capacity (FVC), forced expiratory volume in 1 s (FVE1), and maximal voluntary ventilation (MVV).

### 2.4. Transthoracic Echocardiography (TTE)

Standard TTE examinations were performed combined with CPET to measure peak pulmonary arterial pulse wave velocity (PAV) and ejection fraction (EF). The primary approach used during the evaluation of PS severity was a continuous Doppler evaluation of flow velocity. Pediatric cardiologists at the Veteran General Hospital of Kaohsiung performed all the examinations using a sector probe with a frequency of >5 MHz, according to the standard measurement methods for pediatric echocardiography outlined by the American Society of Echocardiography [18]. The analysis included the highest velocities and EFs. The values obtained during the first and last examinations were compared.

### 2.5. Statistical Analyses

All analyses were performed using the Statistical Package for the Social Sciences for Windows (version 24.0; IBM Corp., Armonk, NY, USA). Continuous data was expressed as mean ± standard deviation, and categorical variables were presented as absolute numbers or percentages. Before each analysis, normality and homoscedasticity were carefully checked. We used paired *t*-test to compare the basic characteristics and results of the initial examination between the patients with PS and controls. We also compared the results between the first and last exercise tests and echocardiographic examinations in patients with PS by paired *t*-test. Statistical significance was set at *p* < 0.05.

## 3. Results

### 3.1. Patient Characteristics

**Figure 1:**
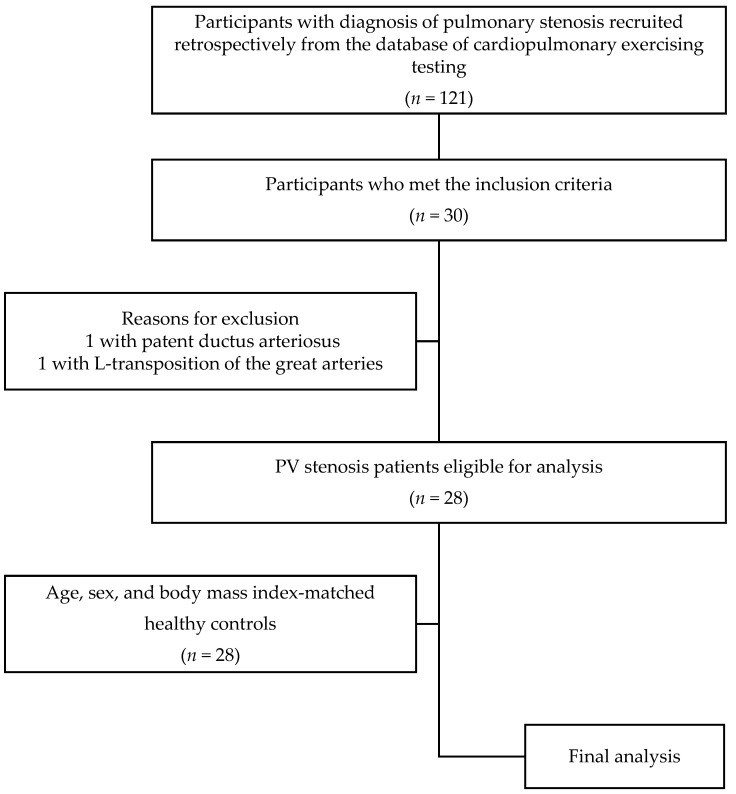
Patient selection process.

A total of 30 patients fulfilled the inclusion criteria; however, two patients were excluded because one had patent ductus arteriosus, and the other had L-transposition of the great arteries. Of the remaining 28 patients, 3 underwent surgery, 16 underwent BPV, and 9 received follow-up care. Moreover, 28 healthy participants with the same age, sex, and BMI as those of the patients were selected as controls. Figure 1 shows the patient selection process.

The average age was 14.50 ± 5.63 years in the control group (39% were girls); the participants were 14.46 ± 6.80 years old at the time of the first CPET and 18.89 ± 8.05 years old at the time of the final CPET (Table 1). No difference in the baseline characteristics was observed between the control and PS groups at the time of the first CPET, except for SBP. Resting SBP was significantly higher in the control group, but the data in both the PS and control groups are within the normal range (Table 2 and Table 3).

### 3.2. Serial CPET and Pulmonary Function Testing in the PS Group

CPET was performed at an average age of 14.46 years in the first CPET and 18.89 years in the last CPET (Table 4). The RER (first CPET: 1.16 ± 0.09, last CPET: 1.18 ± 0.12) indicated that all participants undergoing CPET achieved a maximum level.

There was no significant difference in patient exercise capacity between the first and last examinations, including in AT MET (first CPET: 6.17 ± 1.57, last CPET: 5.91 ± 1.46, *p* = 0.312), peak MET (first CPET: 9.08 ± 2.19, last CPET: 8.77 ± 2.10, *p* = 0.432), peak VO_2_ (first CPET: 31.78 ± 7.68, last CPET: 30.69 ± 7.36, *p* = 0.432), and HRR (first CPET: 28.29 ± 14.65, last CPET: 27.64 ± 11.43, *p* = 0.797) (Table 4). Moreover, the first and last examinations also measured peak HR (first CPET: 173.43 ± 16.42, last CPET: 172.36 ± 13.05, *p* = 0.704), resting HR (first CPET: 85.04 ± 11.85, last CPET: 82.14 ± 11.89, *p* = 0.409), peak systolic blood pressure (first CPET: 168.00 ± 38.37, last CPET: 165.96 ± 29.52, *p* = 0.828), and peak diastolic blood pressure (DBP) (first CPET: 83.29 ± 24.34, last CPET: 90.07 ± 18.68, *p* = 0.232). Additionally, there was no significant difference in pulmonary function between the first and last examinations. However, a declining trend of peak VO_2_% (from 74.20% ± 17.47% to 69.84% ± 16.77%), FVCP% (from 104.47% ± 26.53% to 92.72% ± 13.49%), and FVE1P% (from 103.13% ± 26.13% to 91.34% ± 15.15%) among PS patients was observed. Electrocardiography with exercise revealed no evidence of ischemia, arrhythmia, or continuous VPCs.

### 3.3. TTE

TTE was performed at the same time as CPET. There was no significant difference in PAV between the first and last examinations (first examination group: 1.88 ± 0.45, last examination group: 1.88 ± 0.47, *p* = 0.962). The EF was similar between the first and last examinations (first examination group: 67.46 ± 7.88, last examination group: 69.20 ± 9.46, *p* = 0.556). In addition, our patients were not subjected to impaired RV function. TTE, performed by the pediatric cardiologist, revealed normal sized ventricles without significant impaired RV function. Table 5 presents the results of TTE.

## 4. Discussion

This is the first study to investigate cardiopulmonary function and aerobic fitness in patients with PS through serial CPET and echocardiographic evaluations. This retrospective cohort study revealed that there were no significant differences in peak VO_2_, peak and AT HR, and HRR measured during the first examination between the PS and control groups. Nevertheless, this study found that peak DBP among PS patients was significantly higher than that in the healthy controls. Raised blood pressure (BP) has been commonly recorded as a determining risk factor for stroke and a main risk factor for coronary heart disease [19]. Physicians tend to view diastolic blood pressure (DBP) elevation as more significant than systolic blood pressure (SBP) elevation because it has a closer relation to end-organ damage [20]. DBP is primarily determined based on cardiac output and peripheral vascular resistance. An increase in cardiac output and a decrease in peripheral vascular resistance are influenced by vasodilation of the resistance vessels within the skeletal muscles during exercise. An inappropriately high cardiac output or impaired vasodilation of the resistance vessels within the skeletal musculature causes an increase in DBP during exercise [21]. In our study, we observed a mild decrease in HR without high cardiac output but significantly higher DBP in the PS group. Based on previous studies, it is reasonable to assume that vasodilation of the resistance vessels is poor in this population. Moreover, the AT HR of the PS patients was slightly lower than that of the controls. Von Scheidt et al. [22] detected an impaired chronotropic response in patients with CHD in 2016. Their cohort comprised 103 pediatric patients with CHD, and the peak HR was lower in the CHD group than in the control group. Our findings are consistent with those of the aforementioned studies.

Previous studies have established that patients with PS have lower exercise tolerance. As a case in point, a study on exercise capacity by Goldberg et al. [23] showed that children with PS had a relatively lower maximum endurance time than those who had no cardiac anomalies. Ikkos et al. [24] aimed to find the correlation between working capacity and PV area and found that patients with a PV area index < 0.3 cm^2^/m^2^ had a lower maximum working capacity. They concluded that reduced exercise tolerance in children with PS is linked to decreased stroke volume and cardiac output. In contrast, our study indicated that the exercise capacity of pediatric patients with PS receiving proper management could be comparable to that of healthy individuals. Although disease progression was benign and without a significant increase in PAV among this study’s PS patients, we observed a trend of declining peak VO_2_%, FVCP%, and FVE1P% among these participants after long-term follow-up. With normal growth in children and youngsters, there is a rise in peak VO_2_, pulmonary function, and respiratory muscle strength [25,26,27]. This discrepancy between childhood and adolescence CPET and pulmonary tests findings might be owing to how the body’s physiology at different developmental stages responds to physical activity and the influences of PS on the circulatory system in the long run.

The findings of this study around the encouraging results of proper PS management are consistent with those of previous studies. Voet et al. [28] reviewed cases involving patients undergoing surgery or BPV; they examined the long-term prognosis of treated PS, while patients receiving BPV were followed up for an average of 6 years. They discovered that both surgery and BPV were safe and successful in alleviating TPG. According to the current eligibility and disqualification recommendations for competitive sports for patients with PS, which were proposed by Van Hare et al. [29], competitive athletes with PS who have been treated with surgery or BPV have achieved adequate relief from PS (gradient < 40 mmHg by Doppler evaluation) and can participate in all competitive sports. Nevertheless, these studies were focused on hemodynamic changes instead of a functional assessments of exercise capacity. Patients with CHD are often unaware of their aerobic limitations because they have suffered from abnormal physiology since birth [11]. In 2021, Teng et al. [30] assessed exercise capacity in 84 children with PS by conducting treadmill CPET combined with TTE before and after catheterization and follow-up care; among these children, 43 were treated with BPV, and 41 received follow-up care. The CPET and pulmonary function test results of PS patients treated with BPV and under follow-up care were compared with those of 84 healthy children. Unfortunately, the study provided insufficient longitudinal data. By performing a comprehensive cardiopulmonary assessment, our study directly measured exercise capacity, and based on long-term follow-up data, it has provided further evidence indicating that PS patients can confidently engage in moderate-to-vigorous physical activity since their peak exercise capacity is much higher than that of the minimum recommended vigorous-intensity physical activity levels according to this study’s observations; however, they should receive regular reevaluation to guarantee optimal health. In addition, there are certain limitations associated with the data on exercise capacity in pediatric patients who were treated with BPV or who received follow-up care; thus, it is difficult to find any data on exercise capacity of pediatric patients treated with surgical valvotomy.

Moreover, previous studies have shown that the reasons for diminished physical activities are multifactorial and could be linked to restrictions imposed by concerned parents [31,32]. The findings in this study have shed new light on the exercise capacity of children with PS and provided convincing data that may address parents’ concerns and encourage them to motivate their children to engage more in physical activities. After proper management and cardiac follow-up care, children with PS might be able to engage in physical education classes at school without worrying that they are inferior to their peers.

The scope of this study was limited in some aspects. First, it is open to bias owing to its retrospective nature, and the timing of follow-up varied because of poor follow-up compliance in the real world. Second, the test termination criteria that were adopted were based on the ACSM guidelines [12]. However, we found that there is a lack of studies that have formulated criteria for achieving VO_2_ max when performing exercise testing in children with CHD. Third, we recruited the control group through an age-, sex-, and BMI-matching method, but differences in body composition might have existed between the PS and control groups. Fourth, this study was performed at a single center and had a relatively small sample size; thus, the results might not apply to the entire country’s population. Further research should be performed with large-scale studies involving a wider range of patients in order to shed more light on the effects of PS.

## 5. Conclusions

In this study, based on an adequate evaluation, the exercise capacity of patients with properly managed PS was found to be comparable to that of healthy individuals. PS patients may confidently engage in moderate-to-vigorous physical activity since their peak exercise capacity is much higher than that of the minimum recommended vigorous-intensity physical activity levels according to the observations made in this study. Despite the encouraging long-term outcomes observed in this study, during follow-up, a decreasing trend was discovered in pulmonary function, aerobic metabolism, and peak exercise load capacities among adolescents with PS. Thus, we recommend that PS patients regularly receive reevaluation to guarantee optimal health. This study offers insights that provides the basis for performing further larger cross-national or multicenter studies on PS patients.

## Figures and Tables

**Table 1:** Demographic characteristics of patients with pulmonary valve stenosis.

	First CPET	Last CPET	*p*-Value
Sex (male:female)	17:11	17:11	N/A
Age (years)	14.46 ± 6.80	18.89 ± 8.05	N/A
Height (cm)	150.03 ± 21.17	160.23 ± 16.65	N/A
Body weight (Kg)	47.94 ± 17.66	58.70 ± 17.39	N/A
BMI (Kg/m^2^)	20.46 ± 3.64	22.32 ± 4.27	0.001 *
Body fat (%)	20.72 ± 8.43	22.31 ± 9.51	0.186
Resting SBP (mmHg)	110.07 ± 15.02	120.32 ± 12.75	0.004 *
Resting DBP (mmHg)	66.04 ± 11.80	70.86 ± 8.55	0.083
Resting HR (bpm)	85.04 ± 11.85	82.14 ± 11.89	0.409

BMI, body mass index; DBP, diastolic blood pressure; SBP, systolic blood pressure; HR, heart rate; * *p*-value < 0.05.

**Table 2:** Patient demographic characteristics.

	PS Group First CPET	Control Group	*p*-Value
Sex (male:female)	17:11	17:11	N/A
Age (years)	14.46 ± 6.80	14.50 ± 5.63	0.981
Height (cm)	150.03 ± 21.17	156.39 ± 14.97	0.240
Body weight (Kg)	47.94 ± 17.66	49.90 ± 14.31	0.660
BMI (Kg/m^2^)	20.46 ± 3.64	20.15 ± 3.51	0.750
Body fat (%)	20.72 ± 8.43	19.22 ± 8.79	0.620
Resting SBP (mmHg)	110.07 ± 15.02	121.00 ± 18.32	0.020 *
Resting DBP (mmHg)	66.04 ± 11.80	69.39 ± 12.49	0.295
Resting HR (bpm)	85.04 ± 11.85	83.14 ± 18.31	0.652

BMI, body mass index; DBP, diastolic blood pressure; SBP, systolic blood pressure; HR, heart rate; * *p* value < 0.05.

**Table 3:** Comparison of cardiopulmonary testing and pulmonary function testing in the first examination.

	PS Group	Control Group	*p*-Value
AT MET	6.17 ± 1.57	6.22 ± 1.32	0.899
AT HR (bpm)	135.26 ± 14.10	139.85 ± 14.61	0.326
Peak VE (L)	42.31 ± 12.40	47.30 ± 14.95	0.150
Peak MET	9.08 ± 2.19	9.27 ± 1.78	0.723
Peak HR (bpm)	173.43 ± 16.42	174.86 ± 15.18	0.762
Peak RER	1.16 ± 0.09	1.14 ± 0.08	0.314
Peak SBP (mmHg)	168.00 ± 38.37	158.00 ± 27.56	0.267
Peak DBP (mmHg)	83.29 ± 24.34	72.79 ± 12.36	0.043 *
ΔSBP (mmHg)	57.93 ± 39.28	51.11 ± 37.13	0.452
ΔDBP (mmHg)	17.25 ± 25.02	12.00 ± 18.51	0.338
HRR at 1 min (bpm)	28.29 ± 14.65	24.87 ± 10.83	0.393
Peak VO_2_ (mL/kg/min)	31.78 ± 7.68	32.44 ± 6.23	0.723
FVC (L)	3.26 ± 1.56	2.76 ± 0.94	0.733
FVCP (%)	104.47 ± 26.53	99.62 ± 18.07	0.758
FVE1 (L)	2.72 ± 1.18	2.45 ± 0.85	0.810
FVE1P (%)	103.13 ± 26.13	100.52 ± 14.56	0.563
FEV1/FVC (%)	85.31 ± 9.40	88.41 ± 7.46	0.807
MVV (L)	55.93 ± 19.48	66.98 ± 24.60	0.244
MVVP (%)	91.59 ± 37.76	80.52 ± 20.19	0.078

AT, anaerobic threshold; AT MET, metabolic equivalent at the point of anaerobic threshold; DBP, diastolic blood pressure; HR, heart rate; HRR, heart rate recovery; Peak MET, maximal metabolic equivalent during exercise testing; RER, respiratory exchange threshold; SBP, systolic blood pressure; ΔSBP, the change in systolic blood pressure from rest to exercising; ΔDBP, the change in diastolic blood pressure from rest to exercising; * *p*-value < 0.05.

**Table 4:** Comparison between the initial and final exercising testing and pulmonary function testing data in patients with pulmonary valve stenosis.

	First CPET	Last CPET	*p*-Value
AT MET	6.17 ± 1.57	5.91 ± 1.46	0.312
AT HR (bpm)	135.26 ± 14.10	132.89 ± 14.32	0.406
Peak VE (L)	42.31 ± 12.40	48.83 ± 15.04	0.019 *
Peak MET	9.08 ± 2.19	8.77 ± 2.10	0.432
Peak HR (bpm)	173.43 ± 16.42	172.36 ± 13.05	0.704
Peak RER	1.16 ± 0.09	1.18 ± 0.12	0.455
Peak SBP (mmHg)	168.00 ± 38.37	165.96 ± 29.52	0.828
Peak DBP (mmHg)	83.29 ± 24.34	90.07 ± 18.68	0.232
HRR at 1 min (bpm)	28.29 ± 14.65	27.64 ± 11.43	0.797
Peak VO_2_ (ml/kg/min)	31.78 ± 7.68	30.69 ± 7.36	0.432
Peak VO_2_ (% predicted)	74.20 ± 17.47	69.84 ± 16.77	0.155
FVC (L)	3.26 ± 1.56	3.23 ± 1.05	0.901
FVCP (%)	104.47 ± 26.53	92.72 ± 13.49	0.104
FVE1 (L)	2.72 ± 1.18	2.71 ± 0.77	0.972
FVE1P (%)	103.13 ± 26.13	91.34 ± 15.15	0.143
FEV1/FVC (%)	85.31 ± 9.40	85.02 ± 8.22	0.896
MVV (L)	55.93 ± 19.48	75.80 ± 26.61	0.008 *
MVVP (%)	91.59 ± 37.76	89.45 ± 56.94	0.877

AT, anaerobic threshold; AT MET, metabolic equivalent at the point of anaerobic threshold; DBP, diastolic blood pressure; HR, heart rate; HRR, heart rate recovery; Peak MET, maximal metabolic equivalent during exercise testing; RER, respiratory exchange threshold; SBP, systolic blood pressure; * *p*-value < 0.05.

**Table 5:** Corresponding transthoracic echocardiographic findings of the first and last cardiopulmonary exercise testing in participants with pulmonary valve stenosis.

	First CPET	Last CPET	*p*-Value
LVIDd (cm)	4.16 ± 0.68	4.28 ± 0.61	0.177
LVIDs (cm)	2.47 ± 0.45	2.53 ± 0.39	0.465
LA (cm)	2.15 ± 0.44	2.35 ± 0.56	0.091
AO (cm)	1.89 ± 0.32	2.03 ± 0.39	0.019 *
AOV (m/sec)	1.04 ± 0.10	1.05 ± 0.20	0.723
PAV (m/sec)	1.88 ± 0.45	1.88 ± 0.47	0.962
EF (%)	67.46 ± 7.88	69.20 ± 9.46	0.556

Data are expressed as mean ± standard deviation (95% confidence interval). LVIDd, diastolic left ventricular (LV) internal diameter; LVIDs, systolic left ventricular internal diameter; LA, diameter of left atrium; AO, diameter of aortic root; AOV, peak aortic velocity; PAV, peak pulmonary arterial pulse wave velocity; EF, ejection fraction; * *p* < 0.05.

## Data Availability

The raw data supporting the conclusions of this article will be made available by the authors, without undue reservation.
